# Large Language Models for Summarizing Advance Care Planning Information From Goals of Care Notes in the EHR

**DOI:** 10.1002/lrh2.70086

**Published:** 2026-05-27

**Authors:** Ninad Ekbote, Melody Akhondzadeh, Ross Graham, John Bell, Delee Glasser, Robert El‐Kareh, Andrew Chua, Aaron Boussina, Arin Tai‐Seale, Lily Poursoltan, Karandeep Singh, Parag Agnihotri, Ming Tai‐Seale

**Affiliations:** ^1^ Department of Electrical Engineering UC San Diego San Diego California USA; ^2^ Department of Medicine UC San Diego School of Medicine San Diego California USA; ^3^ Department of Family Medicine UC San Diego School of Medicine San Diego California USA; ^4^ Department of Sociology UC San Diego San Diego California USA; ^5^ Department of Hospital Medicine UC San Diego School of Medicine San Diego California USA; ^6^ Department of Biomedical Informatics UC San Diego School of Medicine San Diego California USA; ^7^ Scripps Green Hospital San Diego California USA; ^8^ Rady School of Management UC San Diego San Diego California USA; ^9^ Population Health Services Organization UC San Diego Health San Diego California USA

**Keywords:** advanced care planning, clinical text mining, large language models, structured data extraction

## Abstract

**Objectives:**

Embedding systematic, structured data extraction within electronic health records (EHR) is vital for improved real‐time insights into care delivery. This study evaluates the feasibility of using large language models (LLMs) to extract structured advance care planning (ACP) information from unstructured Goals of Care (GoC) clinical notes in the EHR.

**Materials and Methods:**

A sample of 100 de‐identified GoC notes was manually annotated by clinicians across four ACP categories: Patient Priorities, Code Status, Decision Maker, and Documentation. Two LLMs (Mistral 24.07 and LLaMA 3.1) were prompted to extract structured outputs without domain‐specific fine‐tuning. Model outputs were compared to human annotations using cosine similarity of BioBERT embeddings.

**Results:**

Mistral 24.07 achieved high semantic similarity in Code Status (0.814), Documentation (0.781), and Patient Priorities (0.770), but lower alignment in Decision Maker (0.609).

**Conclusions:**

LLMs can effectively extract structured ACP information, particularly in well‐documented categories, suggesting potential for scalable, data‐driven feedback loops that improve the provision of care. However, accuracy challenges remain, and further refinement is needed for nuanced qualitative content categories.

## 1 Introduction

Advance care planning (ACP) constitutes discussing and documenting a patient's values, goals, and preferences for future medical care, particularly in the context of serious illness or end‐of‐life situations. ACP is a cornerstone of patient‐centered care, as it improves patient–provider communication and helps ensure that medical decisions align with patient preferences [1]. ACP documentation facilitates goal concordant care in alignment with the patient and family wishes. It has become a goal to have ACP discussions integrated into routine practice so that patients' wishes are recorded in the electronic health record (EHR) and are available when critical decisions arise.

However, there are significant informatics challenges in how ACP information is documented and retrieved in EHRs. One is a lack of standardization; there is no universally adopted place or format for ACP content in most EHR systems, and it often lies fragmented in multiple locations in the EHR [2, 3]. Often, only a small fraction of patients have their ACP preferences recorded in designated structured fields or forms [4], and they are frequently incomplete [5]. Instead, they are often documented within narrative clinical notes [6]. Free text documentation makes it difficult and time‐consuming for clinicians to find the most current ACP instructions, especially when a patient is quickly clinically deteriorating [7, 8].

Informaticians have deployed multiple strategies to improve ACP documentation, such as structured ACP templates [2] and ACP documentation navigators accompanied by clinician training [9]. Uploading scanned ACP documents (e.g., advance directives) is also common, but these documents are often not searchable and are hard to find. Regional initiatives to link ACP registries across healthcare systems suffer from interoperability issues [10]. Because so much ACP data is qualitative narrative data, researchers have attempted to use natural language processing (NLP) to organize ACP data. While rudimentary keyword search and rule‐based algorithms miss crucial details and context [8], more advanced machine learning approaches have managed to successfully identify serious illness conversations from ICU notes [11]. One study achieved high accuracy (F1‐scores 0.84–0.97) detecting whether ACP discussions had been documented when compared to manual chart review of oncology notes [6]. However, NLP typically requires substantial upfront effort to be effective and is prone to failing if clinical language or note styles change. Moreover, these studies focus on identifying the *occurrence* of ACP discussions or documents, rather than reliably *extracting* the content.

More recently, large language models (LLMs) offer a promising avenue for interpreting unstructured ACP documentation. A recent study demonstrated that an LLM approach could accurately identify and classify ACP‐related content for patients with advanced cancer, performing on par with a rule‐based method while requiring far less upfront manual configuration [12]. But LLMs can still misinterpret text or hallucinate, so rigorous validation against clinician‐annotated ground truth is essential [12]. In our pilot study, we evaluated the feasibility of using an LLM to extract structured ACP information from unstructured clinical notes. We focused on Goals of Care (GoC) notes, which are used to document conversations between physicians and patients/surrogates to align treatment with a patient's goals, values, priorities, and prognosis in electronic health records. (see Appendix S1 for an example GoC note). Early evidence suggests LLMs can organize and summarize unstructured GoC data with increasing accuracy [13], requiring increasingly less domain‐specific context [14]. We attempted to identify and summarize four key components of ACP information: (1) Patient Priorities, (2) Code Status, (3) Identified Next Decision Maker, and (4) Patient Completed GoC Documentation. We hope to pilot an ultimately scalable tool that can improve the accessibility of ACP information across the EHR, providing a readily available summary of available ACP information for a given patient.

## 2 Materials and Methods

We used 100 randomly selected and de‐identified GoC notes obtained from the EHR of a large academic medical center, written by attending physicians or residents practicing in Palliative Care, Hospital Medicine, Internal Medicine, Family Medicine, Neurology, Pulmonary and Critical Care, Neurology and Critical Care, and General Surgery. No patient had more than one GoC note included in the pilot. A team of registered nurses manually reviewed each note and coded the content according to four predefined categories, which were deduced through discussions between palliative care specialists, geriatricians, hospitalists, and ACP researchers about what important ACP information should be included in a summary table. These categories are: (1) Patient Priorities—the patient's personal goals, values, or preferences for care; (2) Code Status—the patient's life‐sustaining treatment orders or preferences; (3) Decision Maker—the patient's identified surrogate decision‐maker if they are incapacitated; and (4) Documentation—any completed ACP documentation or forms (e.g., Advanced Directive, Physician Orders for Life‐Sustaining Treatment (POLST) form). Following annotation of the sample GoC notes, the team then discussed and refined the annotation schema and developed an LLM prompting strategy.

Two state‐of‐the‐art open‐source LLMs were selected for evaluation: Mistral 24.07 and LLaMA 3.1. These models were chosen based on their high performance in multi‐turn reasoning and instruction‐following tasks. To simulate real‐world data, no additional fine‐tuning was performed on in‐domain data. Each model was prompted using a zero‐shot (where a model makes predictions without any task‐specific examples, relying entirely on the models' pre‐training to generalize [14]), role‐based prompting strategy that transformed unstructured clinical narratives into machine‐readable data without the use of prior examples, using standardized natural language templates specifically designed to elicit structured outputs corresponding to the four target categories. The prompts were optimized for clarity, specificity, and alignment with the manual annotation schema performed by clinicians. By assigning the LLM a specific persona as a “Clinical Data Extraction Assistant” and providing explicit definitions for the four target Advance Care Planning categories, the prompt established a high‐precision framework for information retrieval. The strategy enforced a strict JSON‐formatted output and included a negative constraint requiring the model to return “Not stated” for missing information, thereby minimizing hallucinations and guaranteeing that the extraction process remained grounded strictly in the provided text. All model training and usage were performed via the AWS Bedrock platform, which offers scalable deployment and access to optimized versions of these LLMs through managed Application Program Interface (APIs). This setup ensures consistency, reproducibility, and performance monitoring during batch processing of clinical notes. While LLaMA 3.1 was included in our initial pilot, it demonstrated less consistency in zero‐shot clinical extraction compared to Mistral 24.07, so it was not used for the full 100‐note evaluation.

To quantitatively assess model performance, we compared LLM outputs to human‐annotated references using a semantic similarity framework [15]. Rather than relying on exact match or string overlap (which penalizes lexical variation), we embedded both the model and human outputs using BioBERT [16], a transformer‐based language model pre‐trained on large‐scale biomedical corpora such as PubMed abstracts and PMC articles. This ensured domain‐relevant semantic representation of medical terminology and clinical phrases. For each category, the cosine similarity between BioBERT embeddings of the LLM‐generated response and its corresponding human annotation was computed. A cosine similarity score closer to 1 indicates high semantic alignment, while scores near 0 indicate low or no similarity. This embedding‐based comparison accounts for linguistic variation and better reflects conceptual agreement. We computed the average cosine similarity for each category individually across the entire dataset, as well as a macro‐averaged similarity score to reflect overall model performance. In addition, we performed qualitative error analysis by reviewing low‐similarity cases to identify systematic failure modes such as hallucinated outputs, category conflation, or omissions. A hospitalist also reviewed an annotated dataset of a sample of 20 GoC notes containing annotations from both the LLM and the initial clinical annotators. Not knowing the identity of the annotator, the hospitalist reviewed GoC note and scored each annotation on a 5‐point Likert scale, with 1 being ‘poor’ annotation quality and 5 indicating ‘excellent’ annotation quality.

## 3 Results

**FIGURE 1:**
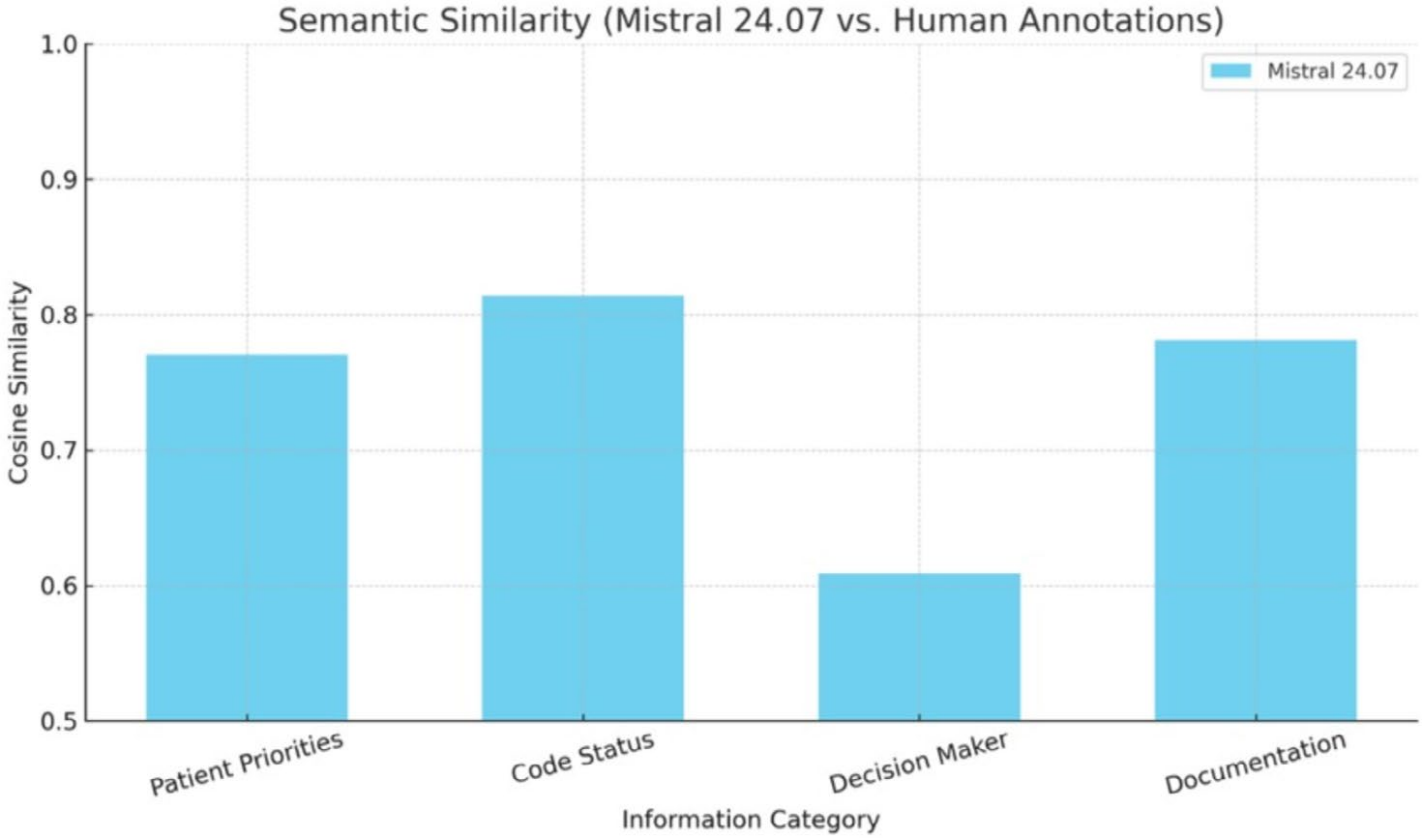
Cosine similarity between Mistral 24.07 outputs and human annotations across four GoC categories.

Using Mistral 24.07, we evaluated the semantic alignment between model‐generated outputs and human annotations across four categories within GoC clinical notes. Cosine similarity, computed using BioBERT embeddings [16], served as the primary evaluation metric. The average cosine similarity scores per category were as follows: Patient Priorities (0.770), Code Status (0.814), Decision Maker (0.609), and Documentation (0.781). These results suggest that the model demonstrates strong alignment in extracting clearly documented clinical concepts (e.g., code status and documentation), but performance drops in more nuanced areas such as decision maker identification (Figure 1). From our randomly selected 20 GoC notes, the assessments by the physician of LLM versus human nurse annotations revealed, on average, that the LLM annotations received a score of 3, while the nurse annotations received a score of 4.

## 4 Discussion

This study demonstrates the feasibility of using LLMs such as Mistral 24.07 to extract clinically meaningful information from unstructured GoC notes. While Mistral 24.07 can approximate expert‐level understanding in structured documentation tasks, additional methods such as prompt tuning or post‐processing may be required for more complex inference tasks involving role identification or implicit contextual cues. The relatively high cosine similarity scores, particularly for Code Status and Documentation, suggest that the model's outputs are semantically close to those generated by clinical experts. These findings underscore the potential of LLMs in supporting automated extraction of ACP components from EHR narratives. However, the comparatively lower performance in the Decision Maker category highlights limitations in the model's ability to resolve role references or infer implicit relationships. This may be due to the subtler linguistic cues involved, such as indirect mentions or varied terminology, which are harder to generalize in zero‐shot settings. We include examples of a GoC note that the LLM struggled to identify a decision maker in the Supporting Information (Appendix S2).

Importantly, our results indicate that the use of foundational LLMs in this domain holds considerable promise but can be further improved. Since our evaluation was conducted on a relatively small sample of notes, this is a proof‐of‐concept requiring intensive further analysis and testing before being deployed over the entire EHR. Generalizability of the LLM approach across different writing and note‐taking styles, clinical scenarios (e.g., oncology vs. geriatrics patients), and languages still need to be established. Since our study used retrospective data, prospective implementation requiring the model to process new notes as they are produced by clinicians can be worthwhile. The model's ability to identify what GoC data is current, and therefore clinically actionable, is critical for widespread implementation in high‐stakes clinical settings. Future evaluation of this approach is necessary. For now, our LLM approach benefits from access to discrete, structured data to build summaries. Structured ACP entries [17], within GoC notes or elsewhere in the EHR, promise wider applicability of LLM summarization.

Future work could explore the impact of alternative prompting strategies—such as chain‐of‐thought prompting (where models must explain their reasoning alongside categorization [18]), few‐shot exemplars (where a model is fine‐tuned or prompted with a few exemplars [14]), or structured question‐answer pairs (in which a model answers predefined questions using constrained response options [19])—to guide the model toward more contextually grounded and complete responses. Additionally, evaluating other instruction‐tuned models such as LLaMA 3.1 or fine‐tuning smaller domain‐specific models may further enhance performance. While LLaMA 3.1 was inferior within the zero‐shot setting of our pilot, its open‐source nature allows for development in a completely isolated environment, promising the ultimate guarantee of data sovereignty. Furthermore, while its zero‐shot performance was lower, LLaMA's architecture is highly conducive to post‐training steps such as domain‐specific fine‐tuning, which we anticipate will significantly improve its efficiency and accuracy in future clinical iterations.

The use of BioBERT‐based cosine similarity as an evaluation metric also proved useful for capturing semantic agreement beyond surface‐level matching. The observed similarity values between LLM and human outputs suggest that these models can serve as reliable assistants in clinical text mining workflows with profound consequences for care outcomes, provided their limitations are well understood and complemented with human oversight. Furthermore, GoC conversations documented in other notes, such as progress notes, could also be incorporated into AI‐assisted summarization. This expansion of source materials could address the challenges of the distributed nature of ACP conversations.

**FIGURE 2:**
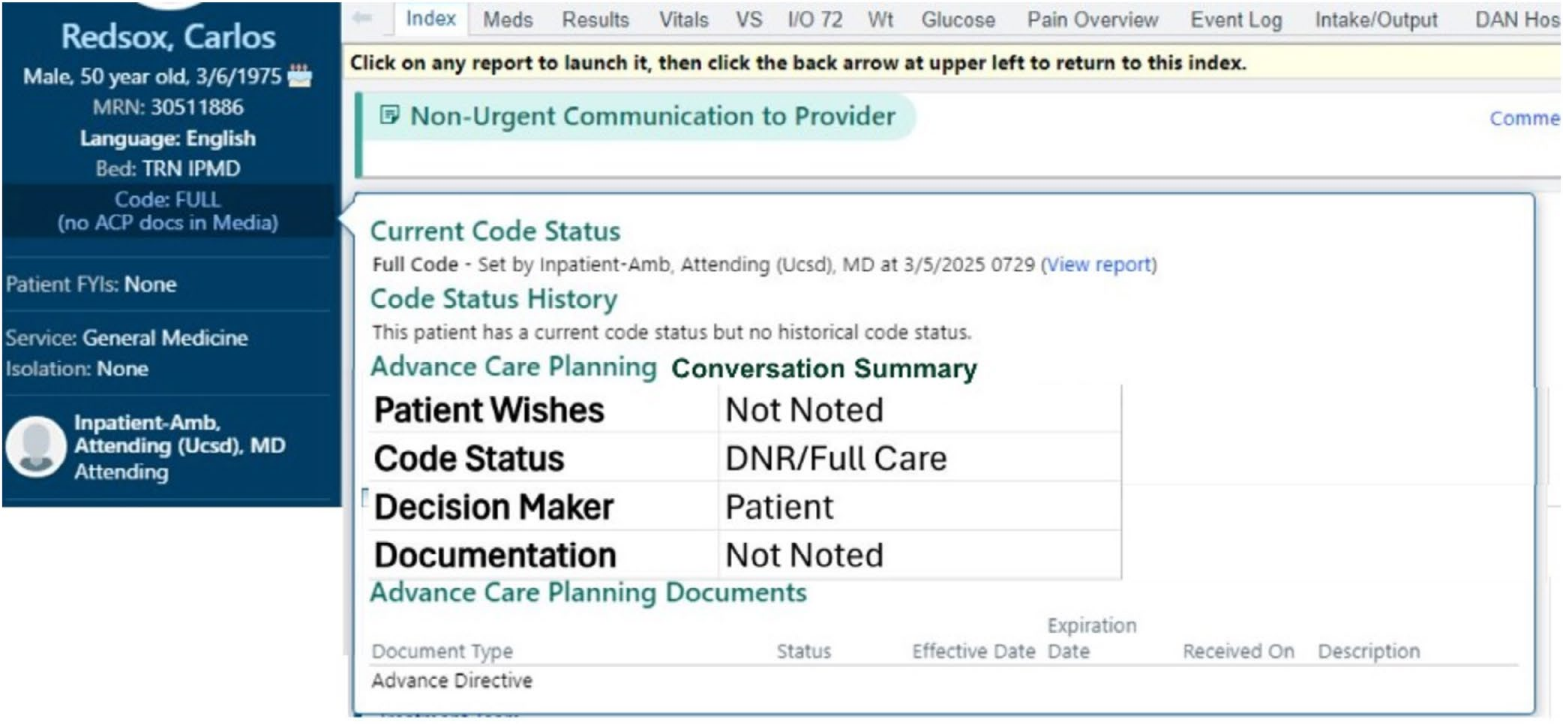
Proposed visualization and location with the EHR.

The study team is considering how to maximize the practical utility of a thoroughly verified LLM prompt from the perspective of a clinician accessing the EHR chart in an urgent clinical context. Figure 2 is a suggested tabular visualization of structured ACP information available on the homepage of the EHR, which displays when the cursor hovers over it. The LLM‐derived structured information should be hyperlinked where appropriate, directing clinicians to the location in the EHR that the LLM drew the information from (e.g., GoC note and Advanced Directive scan), should they require greater detail. We will evaluate the technical feasibility of this suggestion, as well as consulting with clinicians about its practical utility in high‐stake clinical situations. The ability for LLMs to reduce clinicians' time searching through the EHR is as important as the accuracy of the initial summary displayed on the storyboard. Future studies investigating clinician experience using the tool are important next steps.

## 5 Conclusion

Our findings indicate that large language models, particularly Mistral 24.07, are capable of extracting semantically accurate ACP information from GoC notes with a high degree of similarity to human annotations. The model performs well in structured categories such as code status and documentation, with slightly reduced accuracy in complex interpretive categories like decision maker identification. The use of BioBERT‐based embeddings and cosine similarity offers a robust framework for evaluating model outputs in clinical NLP tasks. These results support the viability of LLMs as assistive tools in ACP documentation workflows, while also highlighting opportunities for improvement through advanced prompting techniques and alternative model architectures such as LLaMA 3.1. In time, we anticipate scalable, data‐driven feedback loops that improve the provision of care via real‐time synthesis of EHR information. Future efforts will explore these extensions, with the goal of building scalable, interpretable systems that align with clinical reasoning and documentation standards. Insodoing, we anticipate further improvements to utilizing live patient data in service of improved care and healthcare innovation, a central goal of developing learning health systems.

## Conflicts of Interest

The authors declare no conflicts of interest.

## Supporting information


**Appendix S1:** Example Note #1 & Example Note #2.

**Appendix S2:** GoC Note where surrogate decision‐maker is difficult to identify.

## Data Availability

The data that support the findings of this study are available from the corresponding author upon reasonable request.
