# Supplementary material for: Large Language Models for Summarizing Advance Care Planning Information From Goals of Care Notes in the EHR
Source: Learn Health Syst. 2026 May 27;10(Suppl 1):e70086. doi: 10.1002/lrh2.70086 (PMC13240055; doi:10.1002/lrh2.70086)
Supplement: Supplementary file 1 — Appendix S1: Example Note #1 & Example Note #2. [file LRH2-10-e70086-s001.docx]

**Example Note #1**

At *** on ******(, I approached the patient for a goals of care discussion. I was also accompanied by *************** for the conversation. Previously, despite the patient living with the family, has been independent enough where he went to his own appointments and managed his own medications. When he was told about his cancer and possibly metastasis to his lungs and other organs he did not tell his family as he did not want his family to worry or be alarmed of his medical condition. He was worried that "everyone, including my former students, would know". The fact that the patient was diagnosed with cancer was only revealed to his family until very recently, through his primary care provider. The family noticed the patient becoming more weaker and slower within the past month, without knowing of the patient's medical condition. Talking to the NP who took care of him at his primary care appointment, she referred him to **** due to dramatic decline in strength, hyperglycemia (due to not being able to handle his own medications anymore), mental status change of responding slower and less talkative, and in hopes to establish care with oncology. This morning, the patient was cognitively at his baseline per family, however, the patient remained vague to his family members of any details to his cancer, denied needing help managing his insulin regimen, and has frequently denied any follow up recommended by his primary care provider for oncology or cancer workup. Today, I approached the patient regarding his understanding of his current situation and his goals of care. The patient expressed understanding that he indeed has cancer in his liver that has spread to his lungs and his other organs. He only recently learned about a possible cancer in his prostate, which he denied further workup with urology. When asked about why he has repeatedly denied further workup or follow up appointments, he replied that he has "lived a good life" and that he is "almost 80." He expressed a sense of fulfillment in his life that he taught "hundreds and thousands of students in *****. Some became doctors and I'm so proud of them" and he is happy to have such a big family. He recalls two friends, one who is living with cancer, and another who had cancer but just passed away last year. He concluded by saying "you can't stop what's already done" referring to his cancer metastasis. When asked about his goals in life, he specifically mentions three things. One, to leave a legacy behind. Two, to continue to help others and do good in life. Three, to publish a book, an autobiography. I encouraged the patient to pursue those goals and offered that he would be most successful in achieving these goals if he allowed friends and family to help keep him in good health. He agreed to this and for me to contact his family members regarding his health conditions, help with medication management that is required at home, and assistance in following up with his primary care provider and oncologist. He was very appreciative of our conversation. *****************

**Example Note #2**

Advance Care Planning Patient's Health Care Agent (aka surrogate decision maker):*****************. Confirmed with patient on *******. Patient has the following documents on file: Neither advance directive or POLST. ***** and his family note that he has completed an advance directive in the past. We asked that they bring this to his next in person visit so that it can be uploaded to his medical record. *****************
