# Supplementary material for: Large Language Models for Summarizing Advance Care Planning Information From Goals of Care Notes in the EHR
Source: Learn Health Syst. 2026 May 27;10(Suppl 1):e70086. doi: 10.1002/lrh2.70086 (PMC13240055; doi:10.1002/lrh2.70086)
Supplement: Supplementary file 2 — Appendix S2: GoC Note where surrogate decision‐maker is difficult to identify. [file LRH2-10-e70086-s002.docx]

**Appendix 2: GoC Note where surrogate decision-maker is difficult to identify.**

**Example #1**

Advance Care Planning GOALS OF CARE / ADVANCE CARE PLANNING CONVERSATION NOTE Advance Care Planning

What gives the patient's life meaning? Not assessed patient on video visit with MD Patient would be willing to endure aggressive medical therapies as long as they could still: Not assessed patient on video visit with MD Who would make medical decisions for the patient if they are unable to make decisions for themselves? Not assessed patient on video visit with MD Based on above information I recommended the following: Total time spent face-to-face with patient and/or surrogate decision maker providing counseling related to advance care planning:

*N.B. Approximately 14% of patient notes sampled in our study were notes like this – a template filled in with little or no information. If no information was contained, it was recorded as such by human annotator.*

**Example #2**

GOALS OF CARE / ADVANCE CARE PLANNING CONVERSATION NOTE Advance Care Planning What gives the patient's life meaning? "To fulfill my potential" Major concerns about planning medical care; Accurate information about the issue. Who would make medical decisions for the patient if they are unable to make decisions for themselves? Pt states she has no one to make medical decisions. Total time spent face-to-face with patient and/or surrogate decision maker providing counseling related to advance care planning: 10 minutes
